# Up-Regulation of MUC2 and IL-1β Expression in Human Colonic Epithelial Cells by *Shigella* and Its Interaction with Mucins

**DOI:** 10.1371/journal.pone.0027046

**Published:** 2011-11-04

**Authors:** Radhakrishnan Prakash, Subramaniya Bharathi Raja, Halagowder Devaraj, Sivasitambaram Niranjali Devaraj

**Affiliations:** 1 Department of Biochemistry, University of Madras, Guindy Campus, Chennai, Tamilnadu, India; 2 Unit of Biochemistry, Department of Zoology, University of Madras, Guindy Campus, Chennai, Tamilnadu, India; University of Nebraska Medical Center, United States of America

## Abstract

**Background:**

The entire gastrointestinal tract is protected by a mucous layer, which contains complex glycoproteins called mucins. MUC2 is one such mucin that protects the colonic mucosa from invading microbes. The initial interaction between microbes and mucins is an important step for microbial pathogenesis. Hence, it was of interest to investigate the relationship between host (mucin) and pathogen interaction, including *Shigella* induced expression of MUC2 and IL-1β during shigellosis.

**Methods:**

The mucin-*Shigella* interaction was revealed by an in vitro mucin-binding assay. Invasion of *Shigella dysenteriae* into HT-29 cells was analyzed by Transmission electron microscopy. *Shigella* induced mucin and IL-1β expression were analyzed by RT-PCR and Immunofluorescence.

**Results:**

The clinical isolates of *Shigella* were found to be virulent by a congo-red binding assay. The in vitro mucin-binding assay revealed both *Shigella dysenteriae* and *Shigella flexneri* have binding affinity in the increasing order of: guinea pig small intestinal mucin<guinea pig colonic mucin< Human colonic mucin. Invasion of *Shigella dysenteriae* into HT-29 cells occurs within 2 hours. Interestingly, in *Shigella dysenteriae* infected conditions, significant increases in mRNA expression of MUC2 and IL-1β were observed in a time dependent manner. Further, immunofluorescence analysis of MUC2 shows more positive cells in *Shigella dysenteriae* treated cells than untreated cells.

**Conclusions:**

Our study concludes that the *Shigella* species specifically binds to guinea pig colonic mucin, but not to guinea pig small intestinal mucin. The guinea pig colonic mucin showed a greater binding parameter (R), and more saturable binding, suggesting the presence of a finite number of receptor binding sites in the colonic mucin of the host. In addition, modification of mucins with TFMS and sodium metaperiodate significantly reduced mucin-bacterial binding; suggesting that the mucin-*Shigella* interaction occurs through carbohydrate epitopes on the mucin backbones. Overproduction of MUC2 may alter adherence and invasion of *Shigella dysenteriae* into human colonic epithelial cells.

## Introduction

Mucosal surfaces employ a number of protective strategies to defend against noxious substances and pathogens found within the intestinal lumen. The mucosal surface contains mucins, which are complex glycoproteins synthesized and secreted by epithelial cells of various organs to lubricate and protect luminal surfaces of the human body [1]. However, excessive mucin secretion is a hallmark of the pathogenesis of several diseases, including infectious diseases such as inflammatory bowel diseases and Shigellosis [2]–[4]. The exact role of mucins in gut protection is not completely understood. Although adherence is recognized as an important initial step in other bacterial infections [5]–[7], relatively little information is available on the mechanisms of attachment of *Shigellae* to cells on the mucosal surface. A specific site on the host may be involved in the binding of pathogenic bacteria; for example, *Hemophillus influenzae*, a respiratory pathogen, specifically binds to respiratory mucins but does not bind or cause disease in the intestinal tract. Also, enterotoxigenic *E. coli* infects and causes diseases only in the intestinal tract [8], [9].

Infection of epithelial cells with bacterial pathogens can induce the excessive production of intracellular adhesion molecules (ICAM-1) and mucins (MUC2 and MUC5AC) through the activation of TNFα and Interleukin-1 secretion [10]–[14]. *In vivo* inoculation of *Shigella* species altered the expression of MUC2 and MUC5AC in rabbit intestinal epithelial cells, through production of the inflammatory cytokine TNFα [4]. Proinflammatory cytokines such as TNFα and IL-1β can induce the production of MUC2 and MUC5AC in human intestinal and airway epithelial cells [15]. *Shigella flexneri* stimulates differential mucin gene expression in human colon cancer cells, thereby reducing the production of inflammatory cytokines [16]. The production of different mucins with different levels of expression might involve protection of host epithelium from *Shigella flexneri* induced inflammation [16].

*Shigellae* can induce acute intestinal inflammation through the production of many inflammatory cytokines and chemokines from infected epithelial cells [17]. However, the exact relationships between bacterial infection and types of mucin gene expression under shigellosis have yet to be examined. The present study aimed to examine the interaction between *Shigella* species and intestinal mucin in an attempt to identify region-specific initial binding sites for *Shigella* species. Furthermore, to evaluate the involvement of inflammatory cytokines in the hyper production of MUC2 during shigellosis.

## Methods

### Bacterial strains, media and growth conditions

Clinical isolates of *Shigella dysenteriae* (*S. dysenteriae*) and *Shigella flexneri* (*S. flexneri*) obtained from the Department of Medical Microbiology, Christian Medical College, Vellore, India, were routinely grown in Luria-Bertani (LB) broth or Tryptic soy broth (Himedia, Mumbai, India) at 37°C.

### Isolation and purification of Guinea pig intestinal mucin

The mucin glycoprotein was isolated from Guinea pig as described previously [18]–[20]. Briefly, Male Duncan Hartley strains of guinea pigs were fasted for 48 h and sacrificed by cervical dislocation. The intestine was removed from the duodenal portion after the stomach to the colon, and immediately washed in ice-cold sterile PBS containing protease inhibitors [5 mM EDTA, 1 mM PMSF and 10 mM N-ethyl maleimide (NEM)]. The mucosal surface was gently scraped using a rubber spatula, taking care not to damage the underlying epithelium. The mucus scrapings were lyophilized and redissolved in 50 mM Tris-HCl (pH 8.0) containing 4 M guanidium hydrochloride, 0.02% sodium azide, 1 mM PMSF, 5 mM EDTA and 2 mM N-ethyl maleimide, by stirring overnight at 4°C. The solution was centrifuged at 10,000×g for 20 min at 4°C to remove debris and insoluble material. The mucins were partially purified by Sepharose CL-4B column chromatography. The fractions showing positive reaction with Morgan and Elson assay reagent were pooled, dialyzed and lyophilized. The partially purified mucin samples were stored at −20°C.

### Modification of mucins

Partially purified mucin from Guinea pig small intestine and colon was subjected to various physicochemical treatments and was used for *in vitro* binding studies.

### Tetramethyl urea (TMU) treatment

Treatment of bacteria with Tetra methyl urea was carried out as described in [21]. Briefly, Bacterial cells were incubated with 0.5 M TMU (final concentration) for 1 h at 37°C. Bacteria was harvested by centrifugation, washed to remove excess TMU, and then added to wells coated with mucin.

### Oxidation

50 mg of mucin (dry weight), dissolved in 3 ml of PBS (pH 7.4), was treated with sodium metaperiodate (3.1–100 mM) at 4°C for 1 hr in the dark for nonspecific oxidation of sugar moieties. Oxidized mucin was then dialyzed against distilled water for 24 h and used for binding assays.

### Boiling

Guinea pig colonic mucin (10 mg/ml) was heated in a water bath at 100°C/10 min and cooled to room temperature. These boiled mucins were used for binding assays.

### Partial deglycosylation

Partial deglycosylation of mucin was done as described previously [22]. Guinea pig colonic mucin (∼25 mg dry weight) was treated with 1 ml of TFMS-Anisole reagent at 0°C, on ice. The reaction was terminated by adding a two-fold excess of ether; precooled to 0°C, drop wise, avoiding bubbling due to exothermic heat. The upper layer of ether was removed by centrifugation at 1500 rpm/10 min. the aqueous phase was dialyzed against distilled water to remove traces of ether and pyridinium salts. The dialysate was lyophilized to be used as partially deglycosylated mucin for binding studies.

### Enzyme-linked immunosorbent assay

This assay was done as described in [23]. Briefly, microtitre plates (Nunc, immunomodule Maxisorp Denmark) were coated with serially diluted mucin (150 µg mucin protein/well to 1 µg mucin protein/well) in 0.05 M Carbonate buffer (pH 9.6) and incubated overnight at 4°C. The unbound mucin was removed by three washes with PBS-T (0.05%Tween 20). Residual binding sites were blocked with PBS-2%BSA and incubated at 37°C for 1 h. After removing unbound BSA by three washes with PBS-T, 100 µl containing different concentrations of (cfu/ml) bacteria were added to each well and incubated at 37°C for 1 h. The unbound bacteria were removed by washing three times with PBS-T. 100 µl of mouse monoclonal antibody against *Shigella* species (E-Merck, Mumbai, India) (1∶1000 in PBS-1%BSA) was added to each well and incubated at 37°C for 1 h. The unbound antibody was removed by washing three times with PBS-T. 100 µl of Rabbit anti mouse HRP conjugated secondary antibody (1∶2000 in PBS-1%BSA) was added to each well and incubated at 37°C for 1 h. The unbound antibody was removed by washing three times with PBS-T. Then, the wells were incubated in the dark with 100 µl of substrate (TMB/H_2_O_2_) for 15 min. 100 µl of 1 M H_2_SO_4_ was added to arrest the reaction. The colour developed was measured at 450 nm in a Perkin-Elmer micro plate reader. All experiments were done in triplicate and repeated thrice. Specific binding to mucin (binding parameter ‘R’) was calculated by subtracting the value of bacteria bound to BSA from the values of bacteria bound to mucin. A value of >2 indicates specific binding. Pig gastric mucin was (Sigma, USA) used as a negative control.

### Virulence assay

The virulence nature of *Shigella species* were assessed by Congo-red dye binding assay and the procedure was followed as described previously [24]. Briefly, bacterial strains were grown in Congo red (0.01%) supplemented Tryptic soy broth containing 0.6% yeast extract and 1.5% agar at 37°C for 18 h.

### Cell culture conditions

HT 29 human colon tumor cell line was obtained from National Centre for Cell Science, Pune, India. HT 29 cells were grown in Dulbecco's Modified Eagle Medium (DMEM, GIBCO BRL, Germany), supplemented with 10% Fetal bovine serum (FBS) (Sigma, USA), 100 units/ml Penicillin, 100 µg/ml Streptomycin and 10–20 µg/ml fungisone (Himedia, India).

### Infection of HT 29 cells with *Shigella dysenteriae*

HT 29 cells were seeded into 6 well Costar tissue culture plates at a density of 2×10^5^ cells/ml in volumes of 2 ml per well. At this seeding density, monolayers were sub-confluent (80–90%) at the time of the experiment. Bacteria were grown overnight at 37°C in LB medium and pelleted by centrifugation at 12,000 g for 5 min at 4°C. The cell pellets were washed with PBS (pH 7.4) twice and suspended in antibiotic-free DMEM. Bacteria at 100 cells per epithelial cell (100∶1 ratio) were used to infect for 2 h to allow bacterial entry to occur. Monolayers were washed twice to remove extracellular bacteria and the cultures were incubated for different time intervals in the presence of 50 µg/ml of gentamicin to kill the remaining extracellular bacteria.

### Investigation of bacterial adhesion and invasion of HT 29 cells by Transmission Electron Microscopy (TEM)

Infection of epithelial cells with *Shigella dysenteriae* was done as described above. The infection was stopped at 1 h and 2 h time interval for adherence and invasion analysis by TEM as described previously [25]. Infected epithelial monolayers on culture dishes were fixed in 3% (v/v) glutaraldehyde (in 0.1 M sodium cacodylate buffer, pH 7.4) for 2 h. All the samples were washed in PBS and post fixed in 1% osmium tetroxide for 1 h before dehydration in ethanol and embedded in Epon resin, according to standard procedures. Ultrathin sections (60–100 nm) were cut, and uranyl acetate and lead citrate stains were applied prior to examination and photography with a Philips 201C (Netherland) transmission electron microscope.

### Immunofluorescence studies for mucin gene expression

HT 29 cells infected with *Shigella dysenteriae* were grown for 2 h on cover slips for complete and tight attachment and washed with DMEM in the presence of 50 µg/ml of gentamicin to kill the remaining extracellular bacteria. The infected monolayers were incubated for 0, 1, 3, 6 and 9 h. The infected monolayers were washed with PBS for 5 min and subsequently the cells were permeablized with 0.1% Triton X-100 in PBS for 5 min on ice. Cells were washed and the non-specific binding sites were blocked with PBS containing 0.1% BSA for 10 min at room temperature before antibody incubation. Cells were washed twice with PBS/BSA and incubated with mouse monoclonal antibody for MUC2 at 1∶50 dilution for 1 h at room temperature (E-Merck, Mumbai, India). After the incubation period, monolayers were washed twice with PBS and incubated with secondary antibody (anti-mouse FITC) for 1 h in the dark. After the incubation period, monolayers were washed twice with PBS and the cover slips were mounted with 90% glycerol in PBS. The cells were screened under a fluorescence microscope (Carl Zesis, Germany) with 20× magnifications.

### RT-PCR analysis of Mucin and IL-1β gene expression

Total RNA was extracted from control cells, cells preincubated with actinomycin D (1 µg/ml) for 30 min and *Shigella dysenteriae* infected HT 29 cells, using Trizol reagent (Sigma- Aldrich, USA). For cDNA synthesis, 2 µg of total RNA were used as the template in a 20 µl RT reaction mixture by using Reverse-iT™ first strand synthesis kit (ABgene, UK). 100 ng of cDNA template in a total volume of 25 µl was used for reverse transcription. PCR analysis of mRNAs of MUC2, IL-1β and β-actin were carried out by using forward and reverse primers listed below; for MUC2 gene, 5′- ACAACTACTCCTCTACCTCCA-3′ and 5′-GTTGATCT CGTAGTTGAGGCA-3′; for IL-1β gene, 5′-AAACAGATGAAGTGCTCCTTCCAGG-3′ and 5′-TGGAGAACACCA CTTGTTGCTCCA-3′; for β-actin gene, 5′-GTGGGGCG CCCCAGGCACCA-3′ and 5′-CTCCTTAATGTCCGGACGATTC-3′. The PCR reaction conditions for all the genes were as follows: 94°C for 2 min (1 cycle); 94°C for 30 s, 58°C for 30 s, and 72°C for 1 min (30 cycles); and 72°C for 5 min. The PCR products were subjected to electrophoresis (130 V, constant-voltage field) on a 1% agarose gel equilibrated in Tris-borate electrophoresis buffer containing ethidium bromide (1 mg/ml). Gels were photographed under UV light. The band intensity was measured by using Image J program.

## Results

### Virulent nature of *Shigella*

**Figure 1:**
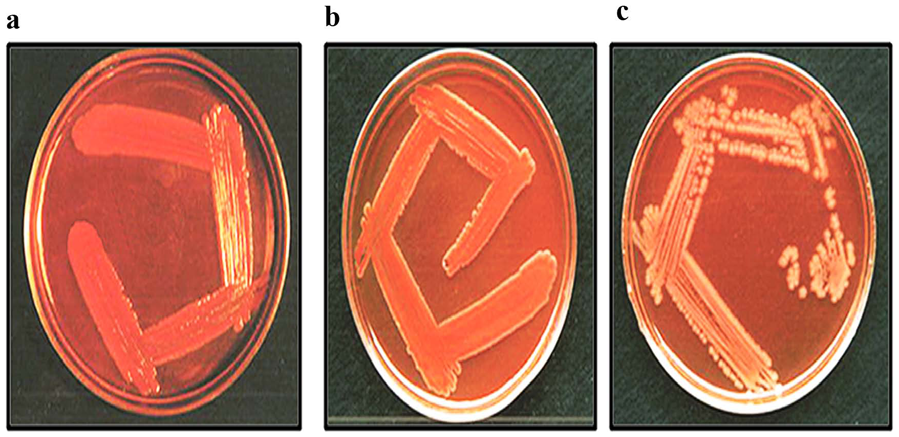
Congo-red binding assay. *Shigella* species were grown in Congo-red agar plate. Orange-pink colour colonies of *Shigella dysenteriae* (a) and *Shigella flexneri* (b) indicate the highly virulent nature and plasmid DNA cured non-virulent *Shigella flexneri* (c) shows white colour colonies.

The virulent nature of the *Shigella* species was analyzed by *in vitro* Congo-red binding assay, appearance of orange/pink color colonies of *S. dysenteriae* (Fig. 1a) and *S. flexneri* (Fig. 1b) suggest that these species are highly virulent when compared to pale white color colonies of plasmid DNA cured non-virulent strain of *Shigella flexneri* (Fig. 1c).

### Bacterial binding to native mucin

**Figure 2:**
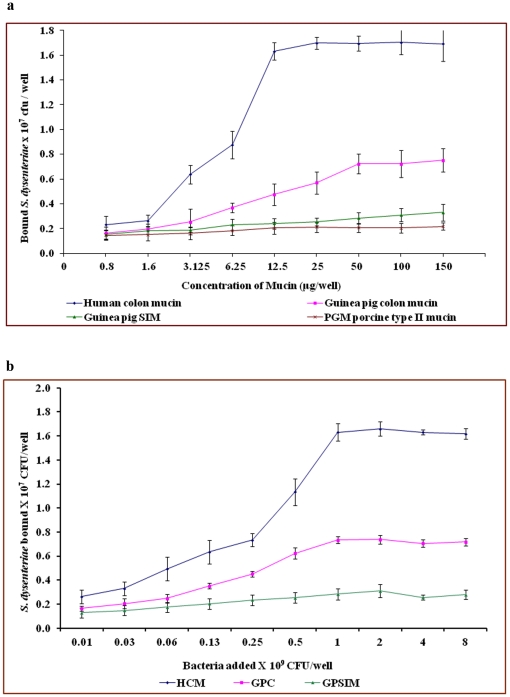
Binding of *Shigella dysenteriae* to mucins. a. Binding of *Shigella dysenteriae* to varying concentrations of different mucins. b. Binding of different amount of *Shigella dysenteriae* to human and guinea pig mucins. Each value is the mean of triplicate determinations. The assay was carried out as described in materials and methods.

**Figure 3:**
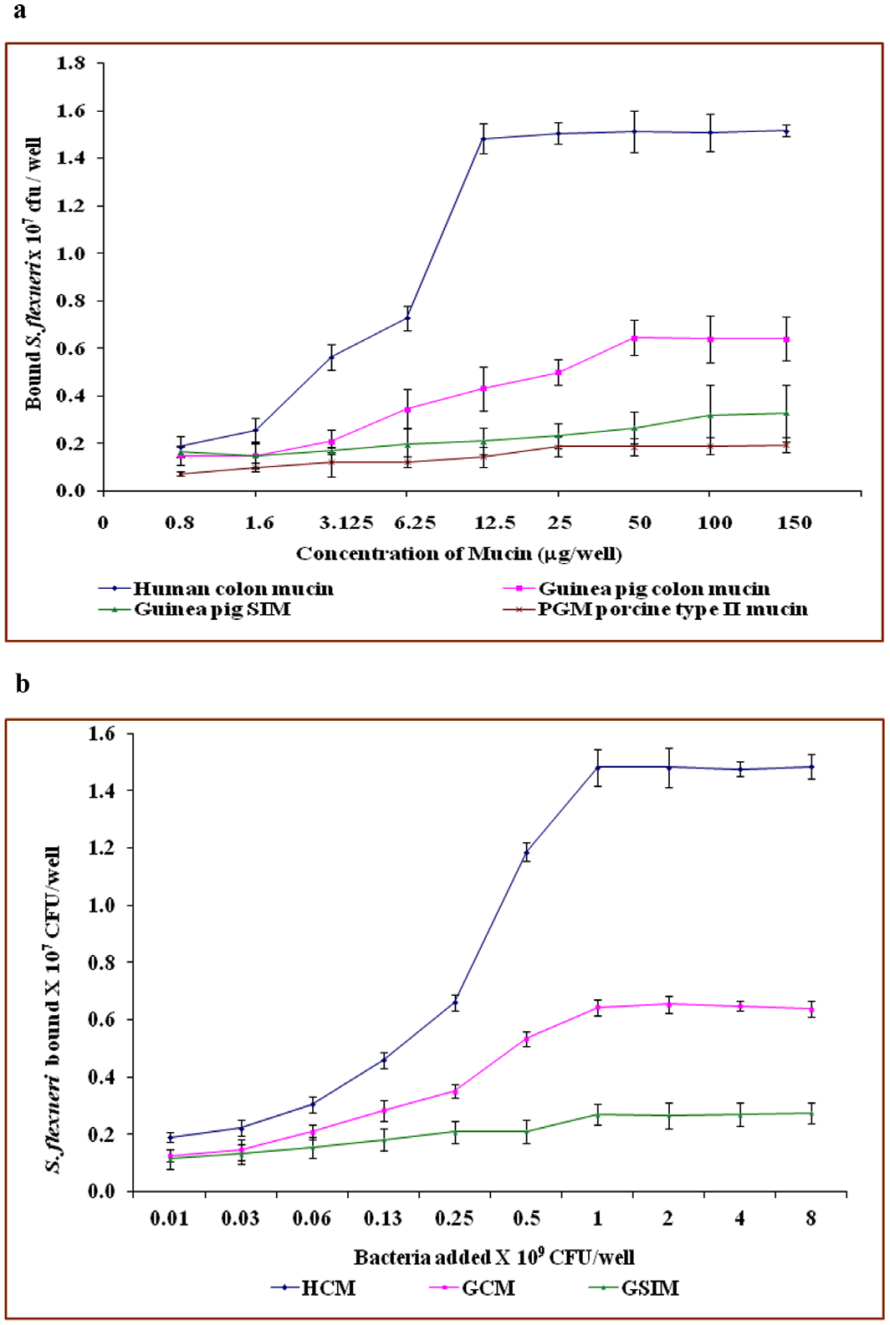
Binding of *Shigella flexneri* to mucins. a. Binding of *Shigella flexneri* to varying concentration of different mucins. b. Binding of different amount of *Shigella flexneri* to human and guinea pig mucins. Each value is the mean of triplicate determinations.

The binding of *Shigella dysenteriae* and *Shigella flexneri* to human colonic mucin and guinea pig colonic mucin was concentration dependent and saturable. Human colonic mucin protein (12.5 µg per well) and Guinea pig colonic mucin protein (50 µg per well) gave sufficient receptor sites (Fig. 2a and 3a). Bacterial concentrations of 1.6×10^7^ cfu/well for *S. dysenteriae* and 1.5×10^7^ cfu/well for *S. flexneri* gave maximal binding to mucins, while bacterial concentrations higher than 1.5×10^7^ cfu/well, the receptor mucin was saturated (Fig. 2b and 3b). The guinea pig small intestinal mucin and Pig gastric mucin concentration of >100 µg/well did not accommodate the same population of bacteria (1.5×10^7^ cfu/well). The number and affinity of binding sites for *S. dysenteriae* and *S. flexneri* was found to be increasing in the order of guinea pig small intestinal mucin (GPSIM) < guinea pig colonic mucin (GPCM) < human colonic mucin (HCM). The binding results suggest a very high degree of site specificity and species specificity. Table 1 represents the binding parameters of *S. dysenteriae* and *S. flexneri* to mucins from different species. None of the clinical isolates bound to unrelated mucin, such as Pig gastric mucin. The human colonic mucin was used as a positive control.

**Table 1:** The binding parameters of *S. dysenteriae* and *S. flexneri* to mucins from different species.

Mucin	Binding parameter ‘R’	
	*S. dysenteriae*	*S. flexneri*
Human colon mucin	7.451±0.326–1.055±0.321	6.769±0.289–0.854±0.19
Guinea pig colon mucin	3.357±0.138–0.753±0.228	2.937±0.129–0.680±0.184
Guinea pig small intestinal mucin	1.415±0.249–0.693±0.182	1.228±0.162–0.746±0.101
PGM porcine type II mucin	0.918±0.18–0.662±0.182	0.868±0.167–0.332±0.046

Microtiter plates were coated with a fixed concentration of mucin (12.5 µg for human; 50 µg for guinea pig). 100 µl of bacteria (∼1×10^10^ cfu/well) was added. The values are the mean ± S.D triplicate determinations of one representative experiment. (PGM, Pig gastric mucin).

### Bacterial binding to modified mucin

**Figure 4:**
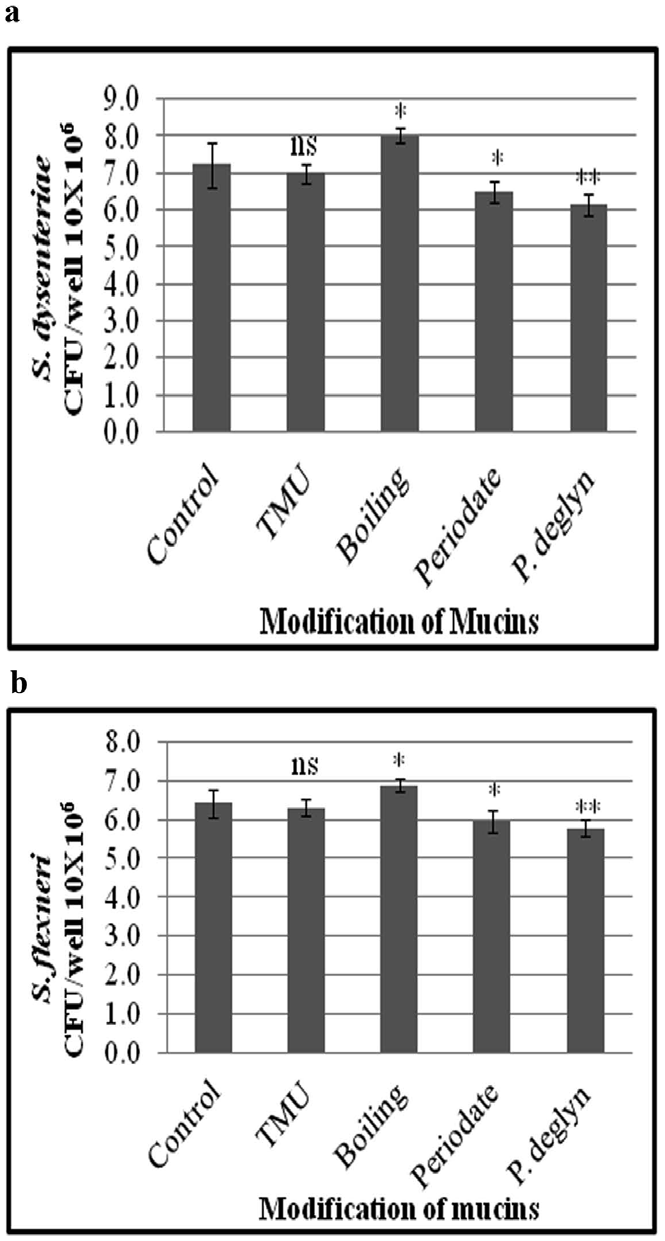
Adherence of *S. dysenteriae* and *S. flexneri* to modified mucins. *S. dysenteriae* (a) and *S. flexneri* (b) showed significantly reduced adherence to sodium metaperiodate and TFMS treated mucins. Whereas, mucins treated with TMU did not affect the bacterial binding to GPCM. Boiling of mucins at 100°C for 10 min significantly increased bacterial binding to GPCM. Each value is the mean of triplicate determinations. A p value of less than 0.05 is considered to be significant. Each comparison was made on bacterial adherence to untreated versus treated mucins.

**Figure 5:**
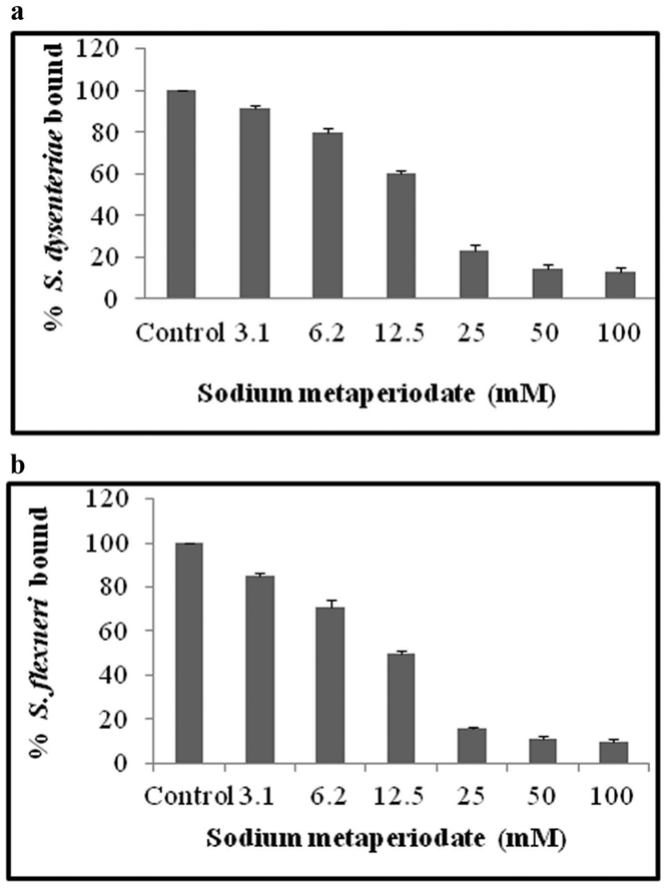
Adherence of *S. dysenteriae* and *S. flexneri* to sodium metaperiodate treated mucins. *S. dysenteriae* (a) and *S. flexneri* (b) showed significantly reduced adherence to sodium metaperiodate treated mucins in a concentration dependent manner when compared with untreated mucins. Each value is the mean of triplicate determinations. A p value of less than .05 is considered to be significant. Each comparison was made on bacterial adherence to untreated versus treated mucins.

The possibility of non-specific hydrophobic interactions mediating the binding to BSA and mucin was investigated by using an effective hydrophobic interaction inhibitor, Tetramethyl urea. Bacterial binding to small intestinal mucin of guinea pig and BSA were diminished at 0.5 M concentrations of TMU (data not shown). However, TMU, did not have any significant effect on *S. dysenteriae* and *S. flexneri* binding to guinea pig colonic mucin. The adherence of *S. dysenteriae* and *S. flexneri* to boiled mucin was significantly higher than the untreated native mucin (Fig. 4a and 4b), whereas, treatments of mucins with periodate and TFMS showed significantly reduced binding to *S. dysenteriae* and *S. flexneri* (Fig. 4a and 4b). Guinea pig colonic mucins that were treated with varying concentrations of periodate show markedly decreased binding to *S. dysenteriae* and *S. flexneri*, in a concentration dependent manner (Fig. 5a and 5b).

### Adhesion and invasion of *Shigella dysenteriae* to HT29 cells by TEM

**Figure 6:**
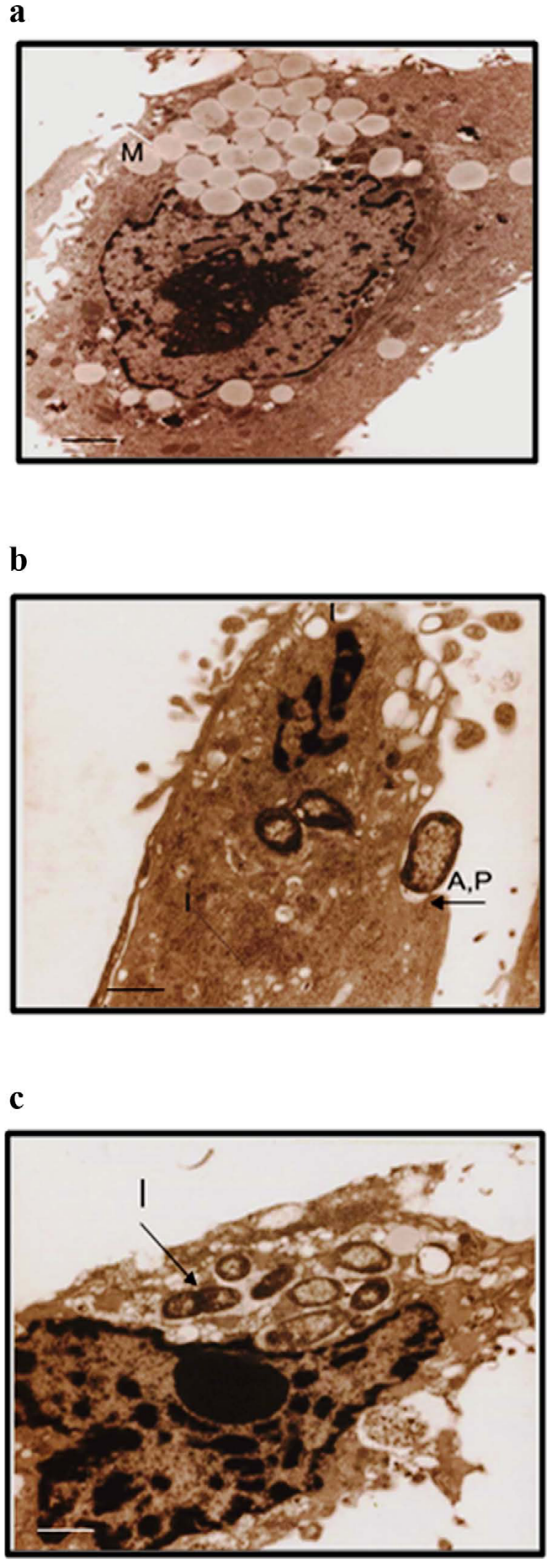
*Shigella dysenteriae* adherence and invasion of human epithelial cells by Transmission Electron Microscopy. After 1 h and 2 h of infection, cells were fixed and processed for Transmission Electron Microscopy analysis as described in materials and methods. Control cells showed intact nuclei and other cell organelles (a). 1 h of infection of HT 29 cells with *Shigellae* showed the adherence and pseudopod projection formation (b). Endocytic processes and phagocytosis of bacteria was seen in the vacuoles of HT 29 cells at the end of 2 h infection (c). The scale bars indicate 1 µm. [M – Mucin; A – Adherence; P – Pseudopod formation; I - Invasion].

The control HT 29 cells showed intact nuclei, cytoplasm and other cell organelles (Fig. 6a). HT 29 cells infected with *Shigella dysenteriae* (1–2 h) showed the intimate contact between the bacterium and host cell membrane (Fig. 6b). Also, endocytic processes, formation of pseudopod projections and phagocytosed bacteria were located in the vacuoles of *Shigella dysenteriae* infected HT 29 cells (Fig. 6c).

### Expression of MUC2 and IL-1β gene in HT 29 cells by RT-PCR

**Figure 7:**
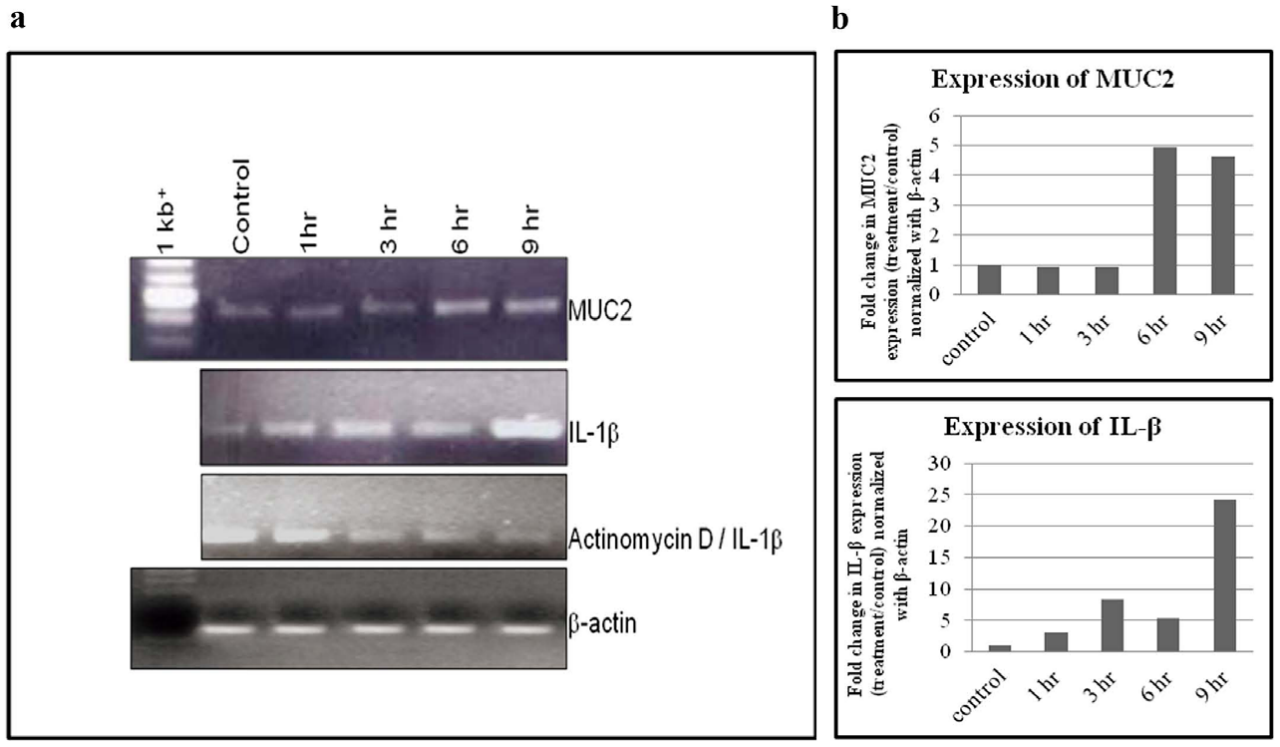
RT-PCR analysis of MUC2 and IL-1β expression in *Shigella dysenteriae* infected HT 29 cells. HT 29 cells were infected with *Shigella dysenteriae* for different time intervals. After infection, mRNA was isolated for MUC2 and IL-1β gene expression analysis as described in methods. HT-29 control cells showed basal level expression of both MUC2 and IL-1β. Whereas, higher level (intensity) expression of both MUC2 (∼4–5 fold higher for 9 h) and IL-1β (25 fold higher for 9 h) were seen in a time dependent manner upon HT-29 cells infected with *S. dysenteriae*. HT 29 cells pre-incubated with Actinomycin D (1 µg/ml) for 30 min, prior to *S. dysenteriae* infection, showed lower level of expression of IL-1β. The band intensity was measured by using image J program. Amplification of β-actin was used as an internal control.

MUC2 (348 bp) and IL-1β (388 bp) gene expressions were observed at basal levels in control cells, whereas, in the *S. dysenteriae* infected conditions, higher levels of MUC2 (∼4–5 fold for 9 h) and IL-1β (25 fold for 9 h) mRNA were observed in a time dependent manner (Fig. 7). Prior to *S. dysenteriae* infection, HT 29 cells were pre-incubated with actinomycin D (1 µg/ml) for 30 min, which showed lessIL-1β expression with increasing time of incubation (Fig. 7). Amplification of β-actin (131 bp) was used as an internal control.

### Immunofluorescence analysis of MUC 2 expression

**Figure 8:**
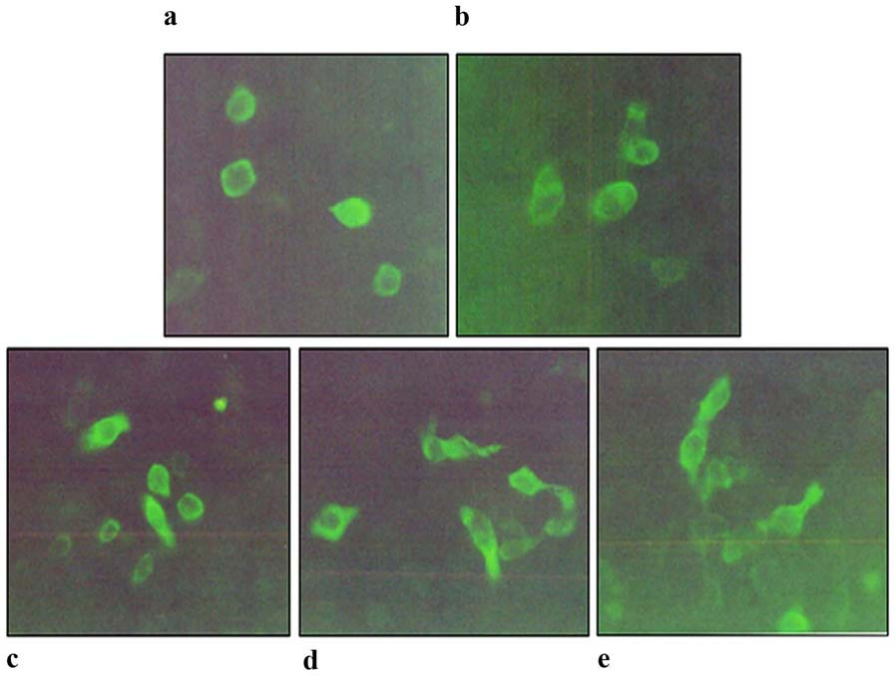
Immunofluorescence analysis of MUC2 expression in *Shigella dysenteriae* infected HT 29 cells. HT 29 cells were infected with *Shigella dysenteriae* in the ratio of 100 bacteria per epithelial cell. Cell monolayers were washed twice to remove extracellular bacteria and the cultures were incubated for different time intervals (0–9 h) in the presence of 50 µg/ml of gentamicin for mucin gene expression analysis. The percentage (%) of cells which expressed MUC2 was classified as follows: −, none; +, light or weak; ++, moderate; +++, intense. In each experiment 300 cells were counted. Control cells, up to 9 h showed a weak signal (+) and for 1 h incubation with *S. dysenteriae* (+); for 3 h, (++); for 6 h, (+++); and for 9 h incubation (+++). (20× magnification).

HT 29 cells were infected with *Shigella dysenteriae* for 2 hours and allowed to incubate at different time intervals (0–9 hours) before analysis of MUC2 expression. Approximately 300 cells were positive with green fluorescence of MUC2 expression. Control HT 29 cells showed normal basal level expression of MUC2 (+) up to 9 h of incubation (Fig. 8a), whereas, HT 29 cells infected with *Shigella dysenteriae* showed increased expression of MUC 2 in a time dependent manner (+, 1 h; ++, 3 h; +++, for 6 and 9 h) (Fig. 8b–e).

## Discussion

The pathogenesis of most of enteropathogens occurs by successful colonization, adherence and invasion on the epithelial cells in the gastrointestinal tract. Previous studies suggest that the invasion of enteropathogens into intestinal epithelial cells is an important step in virulence mechanism of *Shigella* pathogenesis [26]. During the course of infection, bacterial colonization occurs by the interaction of their adhesin(s) to cognate receptors available on epithelial cells or the extracellular matrix [27]. Reports are available on the mechanism of initial attachment by various pathogens like *Vibrio cholerae* [28], *Salmonella typhimurium* [29], and *Yersinia enterocolitica* [30] to the host mucus layer and intestinal epithelial cells.

Earlier work on the *in vitro* binding of *S. flexneri* [31]–[32] and enteropathogenic *E.coli* [33] to intestinal epithelial cells showed that the adherence of the bacterium is due to the presence of plasmid DNA (∼140 MDa) encoded factors. These are known as Invasive plasmid antigens (Ipa). IpaA, IpaB, IpaC, IpaD, IcsA and IcsB, and are essential virulence factors for attachment, invasion and intracellular spread [34]–[36]. The virulent nature of *S. dysenteriae* and *S. flexneri* were determined by Congo red binding on agar plate, where highly virulent *Shigella dysenteriae* showed orange/pink color colonies, suggesting that plasmid DNA and its associated virulence factors are intact, whereas, white color colonies indicate non-virulent bacteria [24].

The mucosa, containing mucin glycoprotein, is thought to have dual roles, one in protecting the host from pathogens, as well as being able to facilitate the preliminary attachment by the pathogens [37]. On the other hand there are reports emphasizing role of mucin glycoproteins in pathogenesis of *Helicobacter pylori* induced gastric carcinoma [38] and *Pseudomonas aeruginosa* infection in cystic fibrosis (CF) patients [39]. To determine whether the mucin layer of the gastrointestinal tract acts as a receptor for *Shigella* species for firm attachment, and if so, is it the protein core or the oligosaccharide structures, which is involved. The virulent *S. dysenteriae* and *S. flexneri* showed preferential binding to human colonic mucin compared with all other mucins. It was clearly evident that the number and affinity of binding sites for *Shigella dysenteriae* and *Shigella flexneri* were found to be increasing in order of guinea pig small intestinal mucin <guinea pig colonic mucin < human colonic mucin. The binding results suggest a very high degree of site specificity and species specificity of *Shigella* species. Similar binding patterns were observed in human colonic mucins with *Shigella dysenteriae* binding [40]. It provides the evidence that the *Shigella* species is able to bind to human colonic mucin with high affinity as compared with guinea pig colonic mucin.

Various pathogens have their own tissue tropism. *Staphylococcus* has a binding affinity to ferret nasal and bovine submaxillary mucin [41]. The peptide backbone of the mucin glycoprotein is unique in some aspects, and shows much more uniformity in profile than do oligosaccharide chains [42]. Diversity of mucin-oligosaccharide structure calls for selective affinity of organisms to mucins of different sources. The Guinea pig colonic mucin (GPCM) may harbor more receptor sites than the others, but less than human colonic mucin receptor sites.

Binding of *Shigella* to guinea pig small intestinal mucin and BSA via the influence of hydrophobic interaction was ruled out, as there was no influence on *S. dysenteriae* and *S. flexneri* binding to TMU treated guinea pig colonic mucin. Thus, the receptor-mediated binding of *Shigella* to GPCM is evident. Further, Mucin pretreated with sodium metaperiodate showed decreased binding, which may be due to oxidation of vicinal hydroxyl groups on carbohydrate moiety [43]. Partial deglycosylation of carbohydrate moieties with TFMS resulted in decreased binding, indicating carbohydrate chains were essential components of the binding sites of GPCM receptor for *Shigella* species. Similar results were identified in many pathogens [44]–[46].

Denaturation of mucins by boiling at 100°C resulted in significant increases the pathogen binding, suggesting that dissociation of the polymer into monomeric glycoproteins exposes a number of new receptor sites for the bacteria. Similar observations were reported earlier with *Yersinia enterocolitica* [47]. It is very clear from this study that *S. dysenteriae* and *S. flexneri* bind specifically to the carbohydrate portion of Guinea pig colonic mucin. Hence, mucin is almost certainly an early site for adherence and establishment of colonization of *Shigella* species.

The pathogenesis of *Shigella* species is mostly contributed by its ability to invade the colonic mucosal epithelium [48]. It still remains unclear whether the initial binding to the colonic mucin would block or facilitate further binding and invasion of *Shigella* to the underlying epithelial cells. Our study shows that most of the bacterial cells entered into the HT 29 cells within 2 h of infection. This invasion was confirmed by transmission electron microscopy and it revealed the occurrence of bacterial adherence, bacteria - induced pseudopod formation, invasion and accumulation in the host cell cytoplasm. This study confirms the previous findings on the invasive nature of *Shigella* species [48].

The relationship between bacterial infection and over expression of MUC2 and MUC5AC were emphasized especially in the respiratory tract and middle ear epithelial cells [11], [49]–[52]. *Pseudomonas aeruginosa*, a common Cystic fibrosis pathogen, activates MUC2 mucin gene transcription by activation of a Src-dependent Ras-MEK1/2-ERK1/2-pp90rsk-NF-κB pathway [53]. Our Previous findings on rabbit ileal loop infection assay, *in vivo*, with *Shigella* species showed up-regulation of MUC2, MUC5AC and proinflammatory cytokine expressions [4]. The present study shows that the expression of MUC2 transcript was increased in a time - dependent manner in HT 29 cells infected with *Shigella dysenteriae*. Further, its expression at protein level was confirmed by immunostaining infected cells with MUC2 antibody. The results revealed a time dependent increase of MUC2 expression. This study suggests that the expression of MUC2 was up-regulated at both transcriptional and translational levels. These observations suggest that mucins are normally expressed in human colonic epithelial cells, but under infectious conditions, are upregulated, and may be involved in the mucociliary clearance of enteric pathogens.

The expression of the proinflammatory cytokine IL-1β was observed in *Shigella* infected macrophages [54]. The major function of IL-1β is to recruit the inflammatory polymorphonuclear cells that infiltrate to the infected site and destabilize the epithelium [55]. In addition to inflammation, it can also induce the expression of MUC2 and MUC5AC mRNA in intestinal and airway epithelial cancer cell lines [56]–[58]. Similar findings were also observed with IL-4, IL-13 and IL-19 on induced mucin gene expression in various epithelial cells [53], [59]–[61]. The present study also confirms the previous reports by showing a time dependent increase in IL-1β and MUC2 expression during *S. dysenteriae* infection of human epithelial cells. Further, IL-1β expression was depleted upon treatment with actinomycin D, which provided additional evidence that *S. dysenteriae* could induce the expression of IL-1β in infected host cells. The over expression of MUC2 may be due to increased expression of IL-1β, consistent with the examples stated above. Further studies are required to identify the mechanism of mucin gene expression and its role in protection against Shigellosis.

## References

[1] Hollingsworth MA, Swanson BJ (2004). Mucins in cancer: protection and control of the cell surface.. Nat Rev Cancer.

[2] Weiss AA, Babyatsky MW, Ogata S, Chen A, Itzkowitz SH (1996). Expression of MUC2 and MUC3 mRNA in human normal malignant and inflammatory intestinal tissues.. J Histochem Cytochem.

[3] Kyo K, Muto T, Nagawa H, Lathrop GM, Nakamura Y (2001). Associations of distinct variants of the intestinal mucin gene MUC3A with ulcerative colitis and Crohn's disease.. J Hum Genet.

[4] Radhakrishnan P, Halagowder D, Devaraj SN (2007). Altered expression of MUC2 and MUC5AC in response to *Shigella* infection and in vivo study.. Biochim Biophys Acta.

[5] Beachey EA (1981). Bacterial adherence: adhesion –receptor interactions mediating the attachment of bacteria to mucosal surfaces.. J Infect Dis.

[6] Duguid JP, Anderson ES, Camphell I (1966). Adhesive properties in Salmonellae.. J Pathol Bacteriol.

[7] Duguid JP, Clegg S, Wilson MI (1980). Adhesive properties of enterobacteriaceae in E.H. Beachey (Ed.), Bacterial adherence..

[8] Davies J, Carlestedt I, Nilssson AK, Hakansson A, Sabharawal H (1995). Binding of *Haemphilius influenzae* to purified mucins from Human Respiratory tract.. Infect Immun.

[9] Mouricout MA, Julien RA (1987). Pillus mediated binding of bovine ETEC to small intestinal mucin.. Infect Immun.

[10] Krunkosky TM, Fischer BM, Martin LD, Jones N, Akley NJ (2000). Effects of TNF-alpha *on expression* of ICAM-1 in human airway epithelial cells in vitro Signaling pathways controlling surface and gene expression.. Am J Respir Cell Mol Biol.

[11] Levine SJ, Larivee P, Logun C, Angus CW, Ognibene FP (1995). Tumor necrosis factor-alpha induces mucin hypersecretion and MUC-2 gene expression by human airway epithelial cells.. Am J Respir Cell Mol Biol.

[12] Dohrman A, Miyata S, Gallup M, Li JD, Chapelin C (1998). Mucin gene (MUC 2 and MUC 5AC) upregulation by Gram-positive and Gram-negative bacteria.. Biochim Biophys Acta.

[13] Smirnova MG, Kiselev SL, Birchall JP, Pearson JP (2001). Up-regulation of mucin secretion in HT29-MTX cells by the pro-inflammatory cytokines tumor necrosis factor-alpha and interleukin-6.. Eur Cytokine Netw.

[14] Zen Y, Harada K, Sasaki M, Tsuneyama K, Katayanagi K (2002). Lipopolysaccharide induces overexpression of MUC2 and MUC5AC in cultured biliary epithelial cells: possible key phenomenon of hepatolithiasis.. Am J Pathol.

[15] Fischer BM, Rochelle LG, Voynow JA, Akley NJ, Adler KB (1999). Tumor necrosis factor-alpha stimulates mucin secretion and cyclic GMP production by guinea pig tracheal epithelial cells in vitro.. Am J Respir Cell Mol Biol.

[16] Nutten S, Sansonetti P, Huet G, Bourdon-Bisiaux C, Meresse B (2002). Epithelial inflammation response induced by *Shigella* flexneri depends on mucin gene expression.. Microbes Infect.

[17] Jung HC, Eckmann L, Yang SK, Panja A, Fierer J (1995). A distinct array of proinflammatory cytokines is expressed in human colon epithelial cells in response to bacterial invasion.. J Clin Invest.

[18] Ringler NJ, Selvakumar R, Woodward HD, Simet IM, Bhavandan VP (1987). Structure of canine trachepbronachial mucin glycoprotein.. Biochemistry.

[19] Devaraj H, Griffith JW, Sheyknazari M, Naziruddin B, Sachev GP (1993). Purification and characterization of monkey (Macca namestrina) tranchobronchial mucin.. Arch Biochem Biophys.

[20] Woodward H, Horsey B, Bhavanandan VP, Davidson EA (1982). Isolation, purification and properties of respiratory mucus glycoproteins.. Biochemistry.

[21] Shuter J, Hatcher VB, Lowy FD (1996). *Staphylococus aures* binding to human nasal mucin.. Infect Immun.

[22] Edge ASB, Faltynek CR, Hof L, Reichert LE, Weber P (1981). Deglycosylation of glycoproteins by Trifluoromethanesulfonic acid.. Anal Biochem.

[23] Tzouvelekis LS, Mentis AF, Makris AM, Spiliadis C, Blackwell C (1991). *In vitro* binding of *Helicobacter pylori* to human gastric mucin.. Infect Immun.

[24] Sasakawa C, Makino S, Kamata K, Yoshikawa M (1986). Isolation characterization and mapping of Tn5 insertions into the 140-megadalton invasion plasmid defective in the mouse Sereny test in *Shigella* flexneri 2a.. Infect Immun.

[25] Tilney LG, Portnoy DA (1989). Actin filaments and the growth movement and spread of the intracellular bacterial parasite Listeria monocytogenes.. J Cell Biol.

[26] Formal SB, Oaks EV, Olsen RE, Wingfield-Eggleston M, Snoy PJ (1991). Effect of prior infection with virulent *Shigella* flexneri 2a on the resistance of monkeys to subsequent infection with *Shigella* sonnei.. J Infect Dis.

[27] Krivan HC, Ginsburg V, Roberts DD (1988). *Pseudomonas aeruginosa* and *Pseudomonas capacia* isolated from cystic fibrosis patients bind specifically to gangliotetraosyl ceramide (asialo GM_1_) and gangliotriaosyl ceramide (asialo GM_2_).. Arch Biochem Biophys.

[28] Freter R, Jones GW (1976). Adhesive properties of Vibrio cholerae - nature of the interaction with intact mucosal surfaces.. Infect Immun.

[29] Embaye H, Batt RM, Saunders IR, Jetty B, Hart CA (1989). Interaction of enteropathogenic *E.coli* O111 with rabbit intestinal mucosa in vitro.. Gastroenterol.

[30] Mantle M, Basaraba L, Peacock SC, Gall DG (1989). Binding of Yersinia enterocolitica to rabbit intestinal brush border membranes, mucus and mucin.. Infect Immun.

[31] Buysse JM, Stover CK, Oaks EV, Venkatesan M, Kopecko DJ (1987). Molecular cloning of invasion plasmid antigen (ipa) genes from *Shigella* flexneri: analysis of ipa gene products and genetic mapping.. J Bacteriol.

[32] Knutton S, Baldini MM, Kaper JB, McNeish AS (1987). Role of plasmid-encoded adherence factors in adhesion of enteropathogenic Escherichia coli to HEp-2 cells.. Infect Immun.

[33] Pal T, Hale TL (1989). Plasmid-associated adherence of *Shigella* flexneri in a HeLa cell model.. Infect Immun.

[34] Nhieu GT, Sansonetti PJ (1999). Mechanism of *Shigella* entry into epithelial cells.. Curr Opin Microbiol.

[35] Basbaum C, Lemjabbar H, Longphre M, Li D, Gensch E (1999). Control of mucin transcription by diverse injury-induced signaling pathways.. Am J Respir Crit Care Med.

[36] Li JD, Dohrman AF, Gallup M, Miyata S, Gum JR (1997). Transcriptional activation of mucin by Pseudomonas aeruginosa lipopolysaccharide in the pathogenesis of cystic fibrosis lung disease.. Proc Natl Acad Sci U S A.

[37] Wells CL, Maddaus MA, Simmons RL (1988). Proposed mechanism for the translocation of intestinal bacteria.. Rev Infect Dis.

[38] Udhayakumar G, Jayanthi V, Devaraj SN, Devaraj H (2007). Interaction of MUC1 with beta-catenin modulates the Wnt target gene cyclinD1 in H. pylori-induced gastric cancer.. Mol Carcinog.

[39] Niranjali Devaraj S, Mostafa S, Stuart W, Bhavanandan VP (1994). Differential binding of Pseudomonas aeruginosa to normal and cystic fibrosis tracheobronchial mucins.. Glycobiology.

[40] Sudha PS, devaraj H, Devaraj N (2001). Adherence of Shigella dysenteriae 1 to human colonic mucin.. Curr Microbiol.

[41] Sanford BA, Thomas VL, Ramsay MA (1989). Binding of *Staphylococci* to mucus *in vivo* and *in vitro*.. Infect Immun.

[42] Forstner JF (1978). Intestinal mucins in health and disease.. Digestion.

[43] Guthrie RD (1962). Periodate oxidation.. Methods of Carbohydr Chem.

[44] Ramphal R, Pyle M (1983). Evidence for mucins and sialic acid as receptors for Pseudomonas aeruginosa in the lower respiratory tract.. Infect Immun.

[45] Viswanath S, Ramphal R (1985). Tracheobronchial mucin receptor for *Pseudomonas aeruginosa* predominance of amino sugars in binding sites.. Infect Immun.

[46] Shuter J, Hatcher VB, Lowy FD (1996). *Staphylococus aures* binding to human nasal mucin.. Infect Immun.

[47] Mantle M, Husar SD (1994). Binding of *Yersinia enterocolitica* to purified, native small intestinal mucins from rabbits and humans involves interactions with the mucin carbohydrate moiety.. Infect Immun.

[48] Parsot C, Sansonetti PJ (1996). Invasion and the pathogenesis of *Shigella infections*.. Curr Topics Microbiol Immun.

[49] Dohrman A, Miyata S, Gallup M, Li JD, Chapelin C (1998). Mucin gene (MUC2 and MUC5AC) upregulation by gram-positive and gram-negative bacteria.. Biochim Biophys Acta.

[50] Lin J, Haruta A, Kawano H, Ho SB, Adams GL (2000). Induction of mucin gene expression in middle ear of rats by tumor necrosis factor-alpha potential cause for mucoid otitis media.. J Infect Dis.

[51] Li JD, Dohrman AF, Gallup M, Miyata S, Gum JR (1997). Transcriptional activation of mucin by Pseudomonas aeruginosa lipopolysaccharide in the pathogenesis of cystic fibrosis lung disease.. Proc Natl Acad Sci USA.

[52] Levine SJ, Larivee P, Logun C, Angus CW, Ognibene FP (1995). Tumor necrosis factor-alpha induces mucin hypersecretion and MUC-2 gene expression by human airway epithelial cells.. Am J Respir Cell Mol Biol.

[53] Li JD, Feng W, Gallup M, Kim JH, Gum J, Kim Y (1998). Activation of NF-kappaB via a Src-dependent Ras-MAPK-pp90rsk pathway is required for Pseudomonas aeruginosa-induced mucin overproduction in epithelial cells.. Proc Natl Acad Sci U S A.

[54] Zychlinsky A, Fitting C, Cavaillon JM, Sansonetti PJ (1994). Interleukin 1 is released by murine macrophages during apoptosis induced by *Shigella* flexneri.. J Clin Invest.

[55] Perdomo OJ, Cavaillon JM, Huerre M, Ohayon H, Gounon P (1994). Acute inflammation causes epithelial invasion and mucosal destruction in experimental shigellosis.. J Exp Med.

[56] Enss ML, Cornberg M, Wagner S, Gebert A, Henrichs M (2000). Proinflammatory cytokines trigger MUC gene expression and mucin release in the intestinal cancer cell line LS180.. Inflamm Res.

[57] Kim YD, Kwon EJ, Kwon TK, Baek SH, Song SY (2000). Regulation of IL-1beta-mediated MUC2 gene in NCI-H292 human airway epithelial cells.. Biochem Biophys Res Commun.

[58] Kim YD, Kwon EJ, Park DW, Song SY, Yoon SK (2002). Interleukin-1beta induces MUC2 and MUC5AC synthesis through cyclooxygenase-2 in NCI-H292 cells.. Mol Pharmacol.

[59] Dabbagh K, Takeyama K, Lee HM, Ueki IF, Lausier JA (1999). IL-4 induces mucin gene expression and goblet cell metaplasia in vitro and in vivo.. J Immunol.

[60] Longphre M, Li D, Gallup M, Drori E, Ordonez CL (1999). Allergen-induced IL-9 directly stimulates mucin transcription in respiratory epithelial cells.. J Clin Invest.

[61] Shim JJ, Dabbagh K, Ueki IF, Dao-Pick T, Burgel PR (2001). IL-13 induces mucin production by stimulating epidermal growth factor receptors and by activating neutrophils.. Am J Physiol Lung Cell Mol Physiol.

